# Microtia, Hearing Disability, and Health Inequity: Socioeconomic Disparities in Rehabilitation and Specialized Care in Ecuador

**DOI:** 10.3390/ijerph23080960

**Published:** 2026-07-25

**Authors:** Fabricio González-Andrade, Fausto Coello, Henry Vásconez

**Affiliations:** 1 Dean’s Office of Postgraduate Health Studies, Faculty of Health and Wellness Sciences, Indoamérica Technological University, Quito 170301, Ecuador; 2 Translational Medicine Unit, Faculty of Medical Sciences, Central University of Ecuador, Quito 170403, Ecuador; fecoellos@gmail.com; 3 School of Health Sciences, San Francisco de Quito University (USFQ), Quito 170901, Ecuador; hvasconez@usfq.edu.ec; 4 College of Medicine, The University of Kentucky, Lexington, KY 40506, USA

**Keywords:** microtia, hearing impairment, hearing rehabilitation, access to care, health inequities, socioeconomic factors, congenital anomalies, sustainable development goal 3

## Abstract

**Highlights:**

**Public health relevance—How does this work relate to a public health issue?**

- Microtia is frequently associated with hearing impairment, yet hearing rehabilitation remains underused, indicating an important gap in access to pediatric audiological care.
- In this tertiary referral sample, microtia may serve as a marker of unmet needs in diagnosis, rehabilitation, and specialized craniofacial care.

**Public health significance—Why is this work of significance to public health?**

- By linking clinical severity, hearing disability, family history, and socioeconomic position, this study highlights how clinical vulnerability and barriers to care may intersect in children with microtia.
- The low use of hearing devices despite frequent hearing impairment underscores preventable risks for language development, education, psychosocial well-being, and long-term disability.

**Public health implications—What are the key implications or messages for practitioners, policy makers and/or researchers in public health?**

- Public health systems should strengthen early hearing screening, timely referral, and equitable access to audiological rehabilitation and specialized craniofacial care.
- Policy makers and researchers should prioritize equity-oriented strategies, including financial protection for hearing devices, improved surveillance, genetic counseling when indicated, and integrated multidisciplinary care for vulnerable populations.

**Abstract:**

**Background:** Microtia is a congenital anomaly of the external ear that is frequently associated with conductive or mixed hearing impairment and potential developmental consequences. Although genetic factors may contribute to some cases, social and economic conditions can influence access to diagnosis, hearing rehabilitation, and specialized care. This study examined clinical severity, hearing disability, and socioeconomic characteristics among patients with isolated and familial microtia evaluated in Ecuador. **Methods:** A cross-sectional study was conducted at a tertiary referral center in Quito, Ecuador. A total of 143 patients with microtia were included. Clinical variables included hearing impairment, hearing device use, microtia severity, associated risk factors, and family history. Sociodemographic variables included age, sex, residence, economic level, and healthcare access. Categorical variables were summarized as frequencies and percentages, and continuous variables as medians with interquartile ranges. Group comparisons were performed using the chi-square test and the Mann–Whitney test. Multivariable logistic regression was used to assess factors associated with hearing device use. Statistical significance was set at *p* < 0.05. **Results:** Hearing impairment was present in 41.78% of patients, with similar prevalence in isolated and familial cases. Hearing device use was reported by 19.86% of the sample. Familial microtia showed a significantly different distribution of right-ear severity, with higher frequencies of grade 1 and grade 4 anomalies, whereas isolated microtia was more commonly grade 3. Significant differences were also observed in sex distribution and economic level. Isolated cases were more frequent among males, whereas familial cases showed a higher proportion of females. Familial microtia was concentrated mainly in the low-middle economic stratum. In multivariable analysis, no factor was independently associated with hearing device use, and confidence intervals were wide, indicating limited precision. **Conclusions:** In this tertiary referral sample, microtia-related disability and access to care appeared to reflect the combined influence of clinical vulnerability, familial aggregation, and socioeconomic disadvantage. The low use of hearing devices despite frequent hearing impairment indicates an important gap between clinical need and rehabilitation. Equity-oriented strategies, including early hearing screening, timely referral, financial protection for assistive devices, and improved access to specialized care, may help reduce unmet needs among patients with microtia.

## 1. Introduction

Microtia is a congenital anomaly characterized by partial or complete underdevelopment of the external ear, with reported prevalence varying widely across populations, from approximately 0.8 to 17.4 per 10,000 live births [1]. Although microtia is often recognized by the visible anomaly of the auricle, its clinical relevance extends beyond the external ear. It may be associated with external auditory canal atresia or stenosis, middle-ear anomalies, and ossicular abnormalities, which can result in conductive or mixed hearing loss. The condition may occur as an isolated anomaly or as part of complex syndromic presentations, including Treacher Collins, Nager, Miller, and Goldenhar syndromes [2].

In addition to structural abnormalities, microtia frequently involves conductive or mixed hearing loss, which compromises language acquisition, cognitive development, and educational performance when health systems fail to ensure early detection and intervention [3]. These consequences extend beyond individual morbidity and generate long-term societal costs, particularly in settings with limited access to rehabilitation services. As a result, microtia represents not only a clinical condition but also a relevant target for early-life public health interventions.

Microtia arises from a multifactorial etiology that reflects the interaction between genetic susceptibility and environmental exposures. Familial clustering and syndromic presentations support a genetic contribution, whereas sporadic cases have been associated with maternal metabolic conditions, intrauterine hypoxia, teratogenic exposures, and adverse socioeconomic environments [4]. Despite advances in craniofacial developmental biology, substantial heterogeneity in clinical presentation persists, limiting the ability to establish robust genotype–phenotype correlations [1]. From a public health perspective, this variability complicates early identification and risk stratification, particularly in low-resource settings. Strengthening surveillance systems and integrating genetic and environmental data remain critical to improving early diagnosis. However, current research has focused predominantly on biological mechanisms, with limited attention to the role of structural and social determinants.

Health inequities play a central role in shaping the distribution, diagnosis, and management of microtia. In Ecuador and across Latin America, health system reforms have expanded nominal coverage, yet disparities in healthcare utilization persist, particularly among rural, low-income, and marginalized populations [5]. Persistent inequalities in access to healthcare for women and children highlight systemic gaps in preventive and specialized services [6]. Population-level evidence further demonstrates substantial inequities in maternal, neonatal, and child health interventions, disproportionately affecting populations living in poverty and indigenous communities [7]. These disparities influence not only disease detection but also access to timely and appropriate care. Consequently, children with congenital conditions in disadvantaged settings often experience delayed diagnosis and reduced access to rehabilitation. These patterns reflect broader structural inequities that extend across the life course.

Socioeconomic position strongly determines access to microtia management and long-term outcomes. Families with greater economic resources are more likely to obtain timely reconstructive surgery and hearing rehabilitation, including bone-anchored hearing aids, whereas disadvantaged households face substantial barriers to care [8]. These barriers include delayed diagnosis, limited access to audiological services, and reduced availability of specialized surgical interventions, which increase the risk of persistent disability [9]. Beyond clinical outcomes, these inequities also affect psychosocial development, educational attainment, and social integration. Children in low-resource settings frequently experience stigma and exclusion, reinforcing cycles of disadvantage [10]. Families in lower socioeconomic strata report greater psychological burden and fewer support systems, further exacerbating health inequities [11]. In contrast, integrated care models in high-resource settings mitigate these outcomes through multidisciplinary approaches and coordinated support systems [12]. The absence of such structures in resource-limited contexts leaves substantial unmet needs and perpetuates disparities in care [13].

Despite growing evidence on the clinical and genetic aspects of microtia, limited research has examined how health system inequalities shape access to diagnosis, hearing rehabilitation, and specialized care in low- and middle-income countries. Existing studies have largely focused on epidemiology or surgical outcomes, with insufficient integration of clinical severity and socioeconomic determinants within a public health framework. This gap limits the ability to design effective, equity-oriented interventions and to identify populations at highest risk of adverse outcomes. Moreover, microtia has not been adequately explored as a potential tracer condition to evaluate disparities in pediatric health systems [14].

Recent evidence from Ecuador has also shown that isolated and familial microtia differ in demographic, clinical, and anatomical patterns, supporting the need to analyze these subtypes separately. In that study, familial aggregation was associated with distinctive clinical characteristics, suggesting that the classification of microtia by family history may provide useful information for clinical assessment, surveillance, and referral planning [15].

This study addresses these gaps by examining the interaction between clinical severity, hearing impairment, and sociodemographic factors in patients with isolated and familial microtia in Ecuador. By integrating clinical and social dimensions, the analysis aims to identify patterns of inequality in access to care and to inform public health strategies that promote early detection, equitable access to rehabilitation, and improved long-term outcomes [14].

## 2. Methods

### 2.1. Study Design

An observational, epidemiological, cross-sectional study was conducted to examine clinical and sociodemographic characteristics among patients with microtia.

### 2.2. Setting

The study was conducted at a tertiary referral center in Quito, Ecuador. This center provides specialized evaluation and management for craniofacial conditions and receives patients from diverse geographic and socioeconomic backgrounds. Given the observational design, the analysis focused on identifying associations rather than causal relationships. The study followed established reporting standards for observational research. Because participants were recruited from a tertiary referral center, the sample may overrepresent patients with access to specialized care and underrepresent rural, Indigenous, and severely underserved populations.

### 2.3. Participants

A total of 143 patients diagnosed with microtia who attended clinical consultations during the study period were included. Participants were recruited consecutively to reduce selection bias; however, the hospital-based design may limit generalizability to the broader population.

### 2.4. Definitions

Isolated microtia was defined as unilateral involvement with a contralateral normal ear. Familial microtia was defined operationally as the presence of at least one affected first-degree relative or two affected relatives across generations, based on reported family history. Because molecular confirmation was not available, this category should be interpreted as suggestive of familial aggregation rather than confirmed genetic inheritance. These definitions were used to distinguish isolated cases from cases with possible familial aggregation. Patients of both sexes and diverse socioeconomic backgrounds were eligible for inclusion. Caregivers provided information for pediatric patients when necessary. All participants met clinical diagnostic criteria established by specialized physicians.

### 2.5. Data Collection

Data were collected through structured interviews and clinical assessments conducted by trained personnel.

### 2.6. Variables

Clinical variables included hearing impairment, hearing device use, microtia severity graded according to standard clinical classification, associated risk factors, and family history. Sociodemographic variables included age, sex, education level, employment status, place of residence, housing conditions, access to basic services, mobility, economic level, and healthcare access. Economic level was self-reported and categorized into four predefined strata: upper-middle, middle, low-middle, and low. Healthcare access was classified according to type of service utilization, including public services, private services, social security, armed forces or police services, or no reported access to healthcare.

Because socioeconomic level was self-reported and no validated socioeconomic index, household income measure, or ethnicity/Indigenous status variable was available, the socioeconomic classification should be interpreted as an approximate indicator of social position rather than a comprehensive measure of structural disadvantage. Data quality checks were conducted to ensure consistency and completeness.

### 2.7. Statistical Analysis

Data were analyzed using RStudio version [4.4.7] and IBM SPSS Statistics version 29. Categorical variables were summarized as frequencies and percentages, while continuous variables were reported as medians with interquartile ranges. Normality was assessed using the Kolmogorov–Smirnov test, and nonparametric tests were applied when appropriate. The Mann–Whitney test was used to compare continuous variables between isolated and familial microtia cases, and the chi-square test was used for categorical variables. Logistic regression models were fitted to evaluate associations between socioeconomic factors and key outcomes, including hearing device use and familial microtia. Results were reported as odds ratios (ORs) with 95% confidence intervals. Statistical significance was set at *p* < 0.05. This analytical approach was used to examine clinical and socioeconomic patterns associated with hearing rehabilitation and microtia subtype.

### 2.8. Ethical Considerations

All participants or their legal guardians provided written informed consent before data collection. Data were anonymized to protect participant confidentiality, and the study complied with the Declaration of Helsinki and Good Clinical Practice guidelines. The study did not involve interventions or experimental procedures. Data were stored securely and made available upon reasonable request. Ethical oversight ensured adherence to international standards for research involving human subjects.

## 3. Results

Hearing impairment was present in 41.78% of patients, with similar frequencies in isolated and familial microtia cases (41.96% vs. 41.18%, respectively; *p* = 0.190) (Table 1). Hearing device use was reported by 19.86% of the total sample. Device use was more frequent among patients with isolated microtia than among those with familial microtia, although the difference was not statistically significant (22.32% vs. 11.76%; *p* = 0.177) (Table 1).

Associated risk factors did not differ significantly between isolated and familial cases (*p* = 0.170) (Table 1). More than half of the patients reported no identifiable associated risk factor. Reported family history was more frequent among familial cases than isolated cases (26.47% vs. 7.14%), but the overall distribution of associated risk factors was not statistically significant between groups.

**Figure 1:**
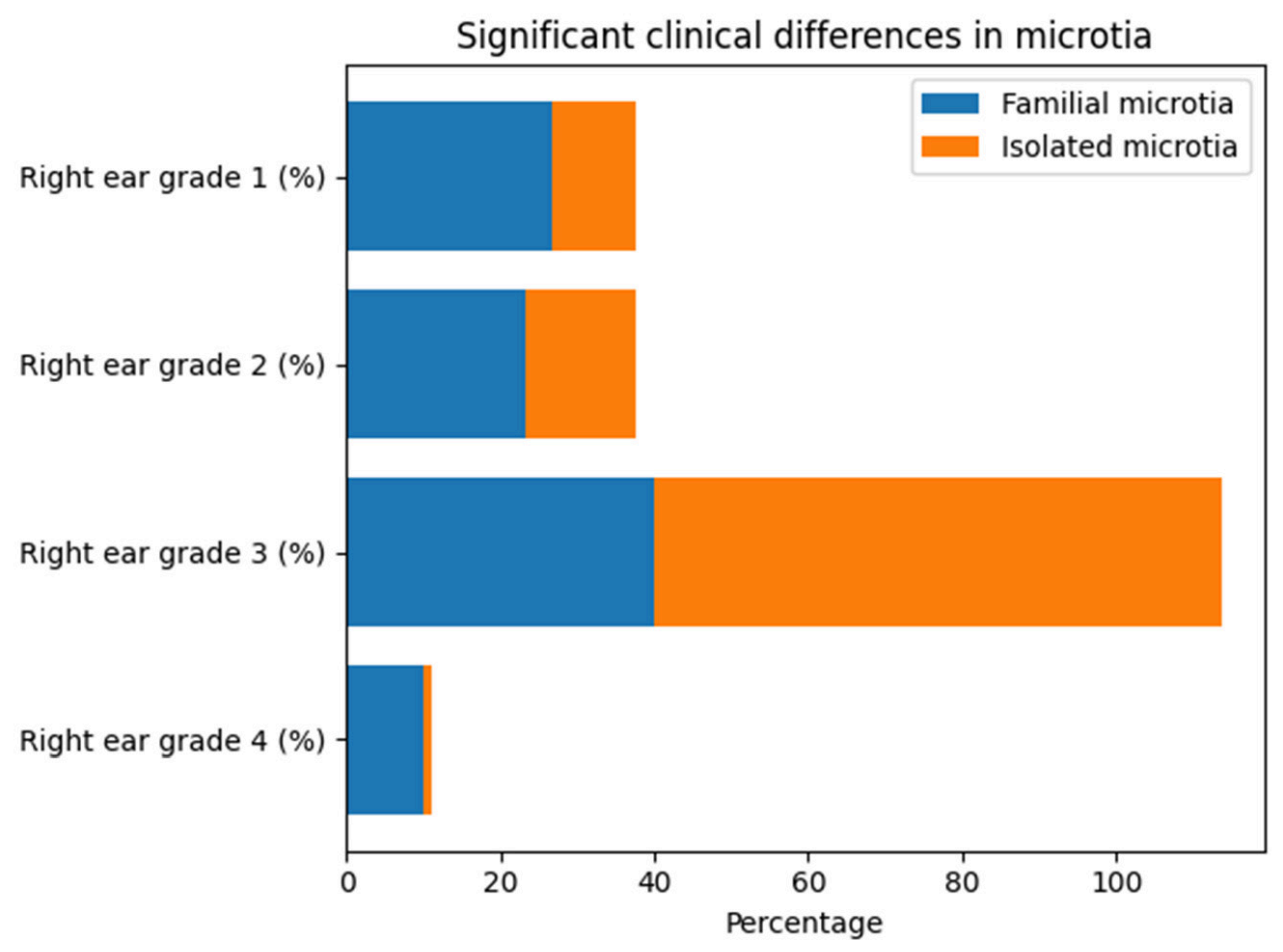
Significant clinical differences in microtia according to right ear severity. Familial microtia shows higher prevalence of grade 1 and grade 4 anomalies, whereas isolated microtia is more frequent in grade 3 cases. Percentages represent the distribution within each group.

Laterality was similar between groups (*p* = 0.934) (Table 1). Right ear involvement was the most frequent pattern in both isolated and familial microtia, followed by bilateral involvement and left ear involvement. Left-ear severity did not differ significantly between groups (*p* = 0.483). In contrast, right-ear severity showed a statistically significant difference between isolated and familial microtia (*p* = 0.003). Grade 3 anomalies were more frequent in isolated cases than in familial cases (73.6% vs. 40.0%), whereas Grade 1 (26.7% vs. 11.0%) and Grade 4 anomalies (10.0% vs. 1.1%) were more frequent among familial cases (Table 1; Figure 1). Hearing status by ear did not differ significantly between groups for either the left ear (*p* = 0.519) or the right ear (*p* = 0.715).

**Figure 2:**
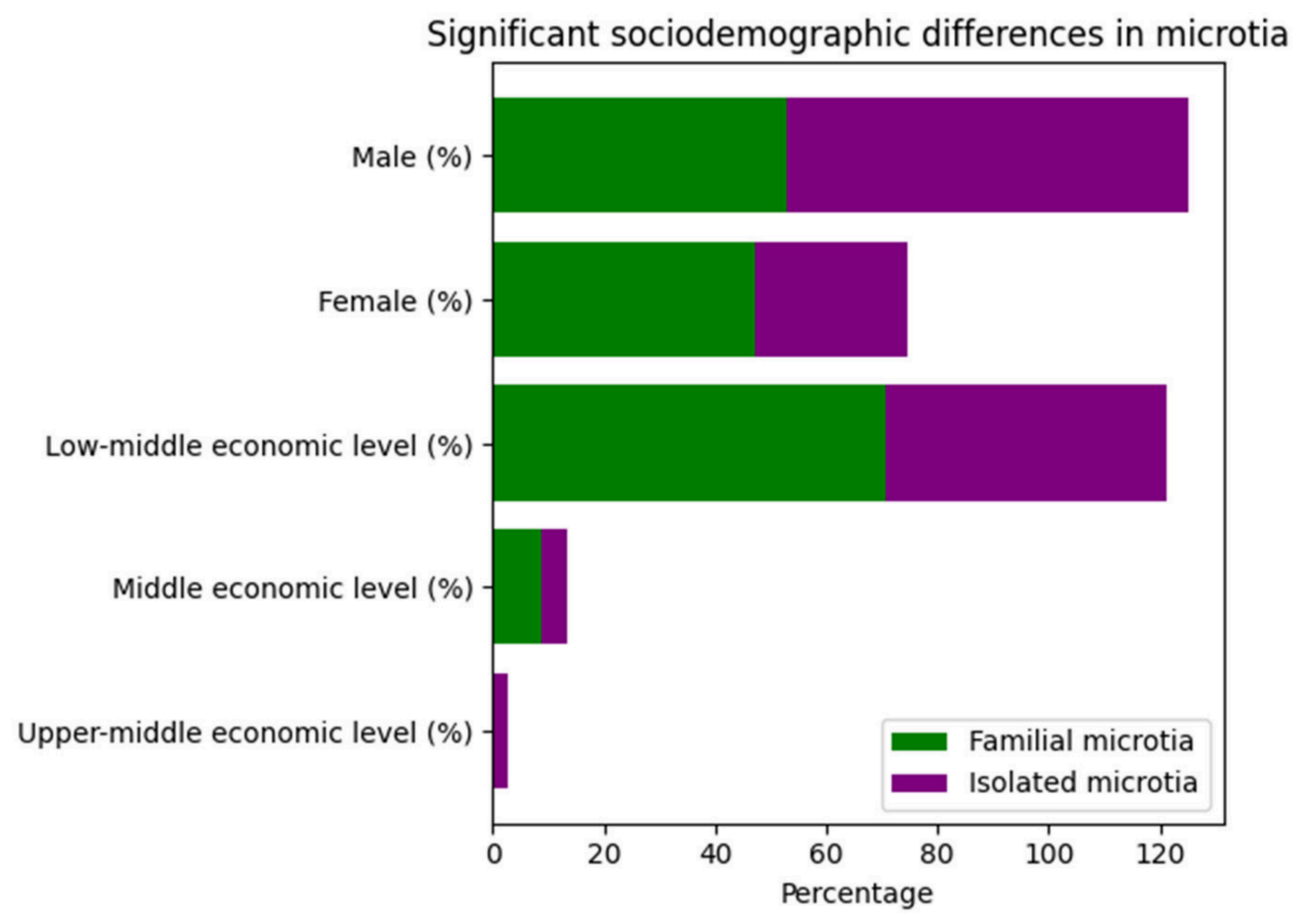
Significant sociodemographic differences in microtia. Isolated cases were more frequent among males and included a higher proportion of patients in the upper-middle economic stratum, whereas familial cases showed a higher proportion of females compared with isolated cases and were concentrated mainly in the low-middle economic stratum.

The median age was 10 years in isolated cases and 11 years in familial cases, with no statistically significant difference between groups (*p* = 0.585) (Table 2). Sex distribution differed significantly by microtia type (*p* = 0.034). Isolated microtia was more frequent among males than females (72.32% vs. 27.68%), whereas familial microtia showed a higher proportion of females compared with isolated cases (47.06% vs. 27.68%) (Table 2; Figure 2).

**Figure 3:**
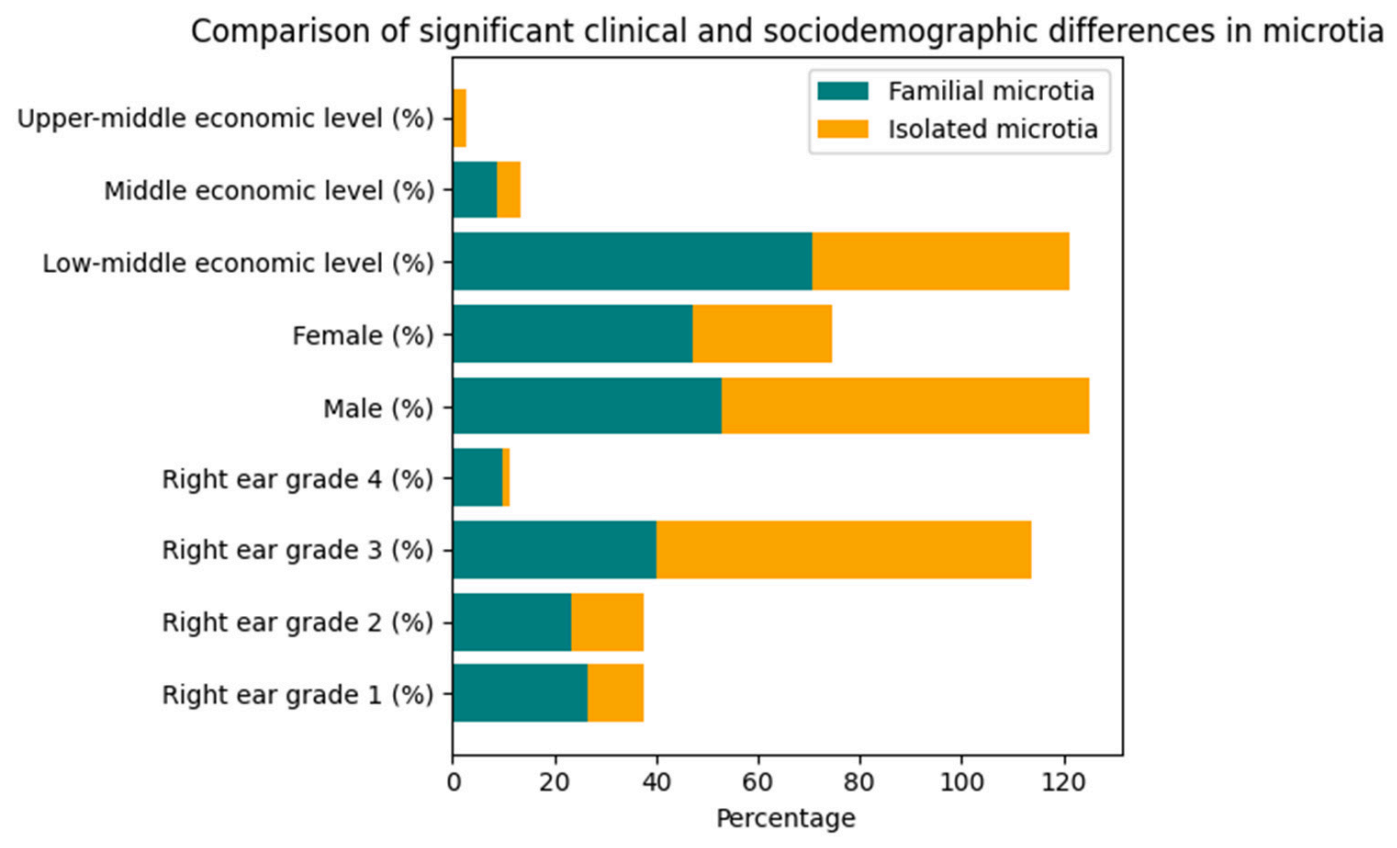
Comparison of significant clinical and sociodemographic differences in microtia. Familial cases showed higher proportions of grade 1 and grade 4 right-ear anomalies and were concentrated mainly in the low-middle economic stratum, whereas isolated cases were more frequent among males and showed a higher proportion of grade 3 right-ear anomalies.

Economic level also differed significantly between groups (*p* = 0.049). Among patients with familial microtia, most were classified in the low-middle economic stratum (70.59%), followed by the low stratum (20.59%) and the middle stratum (8.82%). Among isolated cases, 50.47% were classified as low-middle, 42.06% as low, 4.67% as middle, and 2.80% as upper-middle (Table 2; Figure 2 and Figure 3). Other sociodemographic variables, including education level, employment status, residence, housing type, access to drinking water, internet access, mobility, and healthcare access, did not show statistically significant differences between isolated and familial microtia (Table 2).

**Figure 4:**
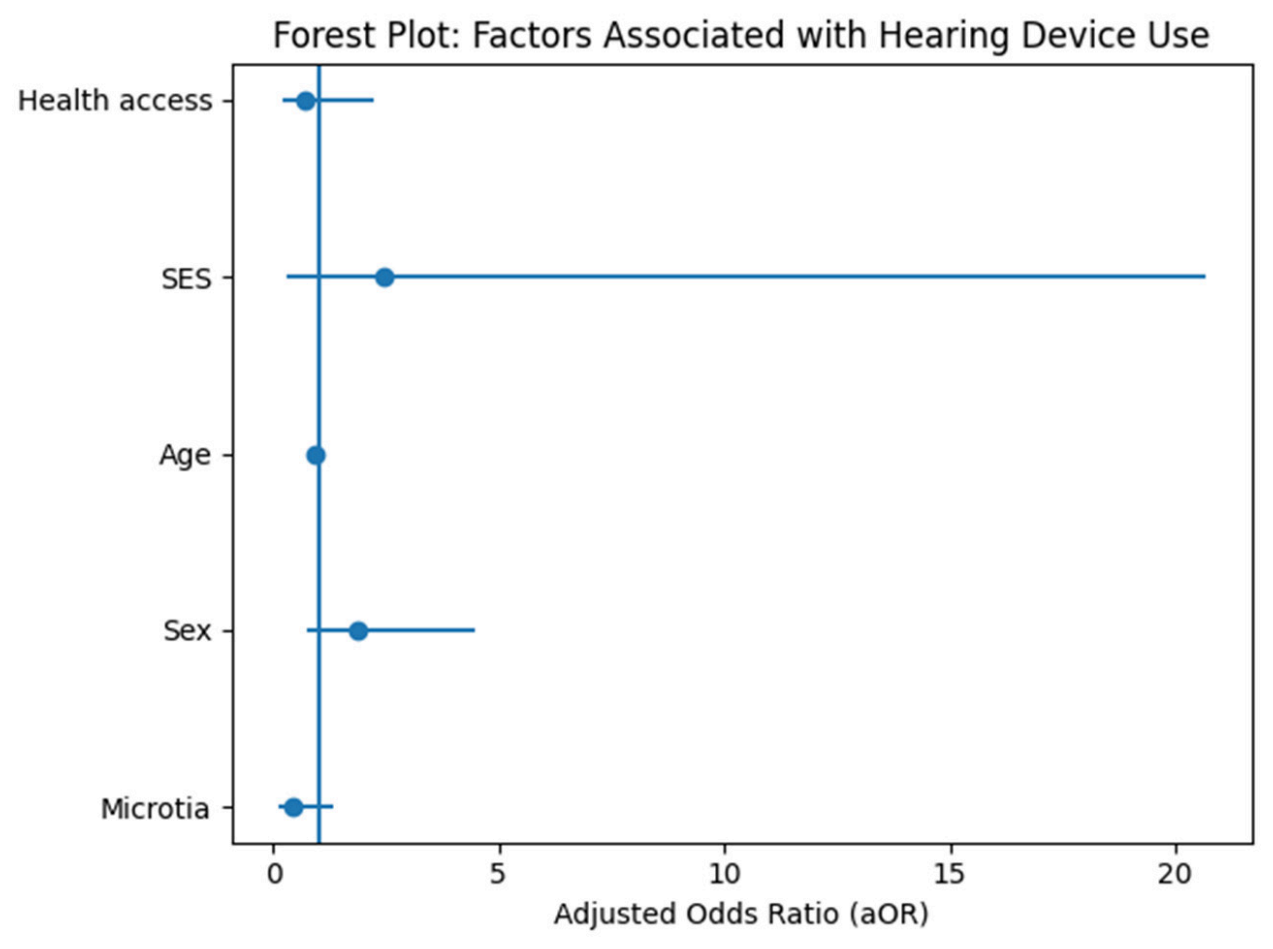
Multivariable logistic regression analysis of factors associated with hearing device use. Forest plot showing adjusted odds ratios (aOR) and 95% confidence intervals for factors associated with hearing device use. The vertical line represents the null value (aOR = 1). None of the variables reached statistical significance; the wide confidence intervals indicate limited precision of the estimates.

In the multivariable logistic regression model, no variable was independently associated with hearing device use (Table 3; Figure 4). Familial microtia was associated with lower odds of hearing device use compared with isolated microtia, but this association was not statistically significant (aOR 0.42, 95% CI 0.13–1.35; *p* = 0.140). Male sex was associated with higher odds of device use, although the confidence interval included the null value (aOR 1.85, 95% CI 0.76–4.49; *p* = 0.170). Age was not significantly associated with hearing device use (aOR 0.91, 95% CI 0.81–1.02; *p* = 0.120). Higher socioeconomic status showed a positive but imprecise association with device use (aOR 2.46, 95% CI 0.29–20.67; *p* = 0.410), while poorer healthcare access showed a negative but non-significant association (aOR 0.69, 95% CI 0.21–2.25; *p* = 0.540). The wide confidence intervals indicate limited precision of the estimates.

## 4. Discussion

In this tertiary referral sample, microtia was associated with a substantial burden of hearing impairment and low use of hearing devices. The findings also showed differences between isolated and familial microtia in right-ear severity, sex distribution, and economic level. These patterns suggest that clinical presentation and access to care may be shaped by both biological and socioeconomic factors. However, because the study was cross-sectional and hospital-based, the results should be interpreted as descriptive associations rather than evidence of causal relationships.

Hearing impairment was common in both isolated and familial microtia, affecting more than 40% of patients. In contrast, fewer than one in five patients reported using hearing devices. This gap between hearing impairment and hearing rehabilitation is one of the most relevant findings from a public health perspective. Children with untreated conductive or mixed hearing loss may experience delays in speech and language development, educational difficulties, and psychosocial consequences. Therefore, the low use of hearing devices in this sample suggests an important unmet need for audiological evaluation, timely referral, and access to rehabilitation.

Clinical severity differed between isolated and familial microtia, particularly for right-ear grading. Familial cases showed proportionally higher frequencies of both grade 1 and grade 4 anomalies, whereas grade 3 anomalies predominated among isolated cases. This finding may indicate phenotypic heterogeneity within familial aggregation. Nevertheless, in the absence of molecular confirmation, familial microtia in this study should be interpreted as a family-history-based clinical category rather than definitive evidence of genetic inheritance. Future studies incorporating genomic analysis, standardized family history assessment, and larger samples are needed to clarify whether specific severity patterns are associated with hereditary mechanisms or syndromic forms.

The observed difference in sex distribution also deserves consideration. Isolated microtia was more frequent among males, whereas familial microtia showed a higher proportion of females than isolated cases. Although this finding was statistically significant, its interpretation should remain cautious. Previous studies have often described male predominance in microtia overall, but sex-specific differences may vary according to phenotype, ascertainment, referral patterns, and family history. In this sample, the difference may reflect biological variation, differential recognition of familial cases, or selection patterns within a tertiary referral setting. Further population-based studies are needed to determine whether sex modifies familial aggregation or clinical presentation.

Economic level differed significantly between isolated and familial cases. Familial microtia was concentrated mainly in the low-middle economic stratum, whereas isolated cases included a higher proportion of patients in the low and upper-middle strata. These findings should not be interpreted as evidence that socioeconomic disadvantage causes microtia. Rather, they suggest that socioeconomic position may influence access to diagnosis, referral, genetic evaluation, hearing rehabilitation, and specialized craniofacial care. In settings where specialized services are centralized or require substantial out-of-pocket expenditure, families with fewer resources may experience delays or discontinuity in care.

The multivariable analysis did not identify statistically significant predictors of hearing device use. Although higher socioeconomic status showed a positive association and poorer healthcare access showed a negative association with device use, the confidence intervals were wide and included the null value. These findings indicate limited precision and suggest that the model may have been underpowered to detect modest associations. Therefore, the regression results should be interpreted cautiously and should not be used as definitive evidence of socioeconomic gradients in hearing rehabilitation. However, the low overall use of hearing devices despite frequent hearing impairment remains clinically and programmatically important.

From a health-system perspective, the findings support the need to strengthen early detection and rehabilitation pathways for children with microtia. Universal newborn hearing screening, timely audiological assessment, referral to specialized craniofacial and otologic services, and financial protection for hearing devices are potential strategies to reduce unmet needs. Multidisciplinary care models that integrate audiology, otolaryngology, plastic surgery, genetics, speech therapy, psychology, and social support may be particularly useful for patients with more severe or familial presentations. In resource-limited settings, decentralization of audiological services and stronger referral networks could improve continuity of care.

This study has several limitations. First, the sample was recruited from a single tertiary referral center in Quito, which may limit generalizability to the broader population of patients with microtia in Ecuador. Rural, Indigenous, and severely underserved populations may be underrepresented. Second, the cross-sectional design prevents causal inference and does not allow assessment of long-term developmental, educational, or rehabilitation outcomes. Third, familial microtia was classified using reported family history, without molecular or genetic confirmation. Fourth, socioeconomic level was self-reported and categorized into predefined strata; no validated socioeconomic index, household income measure, or ethnicity/Indigenous status variable was available. These limitations may have reduced the ability to fully capture structural disadvantage. Finally, the regression model had limited precision, as reflected by wide confidence intervals.

Despite these limitations, the study contributes evidence on the intersection of clinical severity, hearing impairment, family history, and socioeconomic position in patients with microtia evaluated in Ecuador. The findings suggest that microtia can be useful as a tracer condition for identifying unmet needs in pediatric hearing rehabilitation and specialized craniofacial care. Future research should use larger, multicenter, or population-based samples, incorporate validated socioeconomic measures, include ethnicity and Indigenous status where appropriate, and integrate genetic or molecular evaluation when feasible. Such studies would help clarify the mechanisms underlying familial aggregation and support more equitable care strategies for children with microtia.

## 5. Conclusions

Microtia may therefore serve as a useful tracer condition for identifying unmet needs in pediatric hearing rehabilitation and specialized craniofacial care. Strengthening newborn hearing screening, timely audiological assessment, referral pathways, multidisciplinary care, and financial protection for hearing devices could help reduce barriers for vulnerable families. Future multicenter or population-based studies incorporating validated socioeconomic measures, ethnicity, and Indigenous status, and genetic or molecular evaluation are needed to better characterize disparities and inform equitable interventions.

## Figures and Tables

**Table 1:** Distribution of patients by type of microtia according to clinical characteristics.

Features	Total	Microtia		*p*-Value
		Isolated	Familial	
Type of disability (*n* (%))				
Hearing	61 (41.78)	47 (41.96)	14 (41.18)	0.190
Language	1 (0.68)	0 (0)	1 (2.94)	
None	84 (57.53)	65 (58.04)	19 (55.88)	
Use of hearing device (*n* (%))				
Yes	29 (19.86)	25 (22.32)	4 (11.76)	0.177
No	117 (80.14)	87 (77.68)	30 (88.24)	
Has associated risk factor (*n* (%))				
None	81 (55.48)	68 (60.71)	13 (38.24)	0.170
Familial	17 (11.64)	8 (7.14)	9 (26.47)	
Maternal obesity	3 (2.05)	2 (1.79)	1 (2.94)	
Low birth weight	4 (2.74)	2 (1.79)	2 (5.88)	
Twinning	2 (1.37)	2 (1.79)	0 (0)	
Advanced maternal age	8 (5.48)	7 (6.25)	1 (2.94)	
Prematurity	4 (2.74)	3 (2.68)	1 (2.94)	
Exposure to genotoxics	12 (8.22)	9 (8.04)	3 (8.82)	
Low or no folic acid consumption	11 (7.53)	8 (7.14)	3 (8.82)	
Maternal illnesses	4 (2.74)	3 (2.68)	1 (2.94)	
Laterality of the affectation (*n* (%))				
Left ear	29 (19.86)	23 (20.54)	6 (17.65)	0.934
Right ear	71 (48.63)	54 (48.21)	17 (50)	
Both	46 (31.51)	35 (31.25)	11 (32.35)	
Degree of microtia (*n* (%))				
Left ear				
Grade 1	16 (21.33)	12 (20.34)	4 (25)	0.483
Grade 2	12 (16)	11 (18.64)	1 (6.25)	
Grade 3	47 (62.67)	36 (61.02)	11 (68.75)	
Right ear				
Grade 1	18 (14.9)	10 (11)	8 (26.7)	0.003 *
Grade 2	20 (16.5)	13 (14.3)	7 (23.3)	
Grade 3	79 (65.3)	67 (73.6)	12 (40)	
Grade 4	4 (3.3)	1 (1.1)	3 (10)	
Normal hearing (*n* (%))				
Left ear				
Normal	45 (30.82)	33 (29.46)	12 (35.29)	0.519
Low	101 (69.18)	79 (70.54)	22 (64.71)	
Right ear				
Normal	69 (47.26)	52 (46.43)	17 (50)	0.715
Low	77 (52.74)	60 (53.57)	17 (50)	

Note: Chi-square test. * Significant differences. Source: FCM-UCE, own elaboration.

**Table 2:** Distribution of patients by type of microtia according to social characteristics and health disparities.

Features	Total	Microtia		*p*-Value
		Isolated	Familial	
Age years (median (IQR))	10 (8–14)	10 (8–14)	11 (8–14)	0.585
Sex (*n* (%))				
Men	99 (67.81)	81 (72.32)	18 (52.94)	0.034 *
Women	47 (32.19)	31 (27.68)	16 (47.06)	
Education (*n* (%))				
First level	40 (27.4)	30 (26.79)	10 (29.41)	0.254
Second level	68 (46.58)	56 (50)	12 (35.29)	
Third level	38 (26.03)	26 (23.21)	12 (35.29)	
Employment estatus (*n* (%))				
Employee with appointment	14 (9.59)	12 (10.71)	2 (5.88)	0.712
Hiring	45 (30.82)	35 (31.25)	10 (29.41)	
Occasional	29 (19.86)	23 (20.54)	6 (17.65)	
Not working	58 (39.73)	42 (37.5)	16 (47.06)	
Location of housing (*n* (%))				
Urban	104 (71.23)	79 (70.54)	25 (73.53)	0.736
Rural	42 (28.77)	33 (29.46)	9 (26.47)	
Type of housing (*n* (%))				
Own	83 (56.85)	65 (58.04)	18 (52.94)	0.599
Rent	63 (43.15)	47 (41.96)	16 (47.06)	
Access to drinking water (*n* (%))				
Yes	139 (95.21)	107 (95.54)	32 (94.12)	0.665
No	7 (4.79)	5 (4.46)	2 (5.88)	
Access to electricity (*n* (%))				
Yes	146 (100)	112 (100)	34 (100)	-
Internet access (*n* (%))				
Yes	138 (94.52)	106 (94.64)	32 (94.12)	1.000
No	8 (5.48)	6 (5.36)	2 (5.88)	
Available mobility (*n* (%))				
Public transport	109 (74.66)	82 (73.21)	27 (79.41)	0.467
Private transport	37 (25.34)	30 (26.79)	7 (20.59)	
Economic level (*n* (%))				
Upper middle	3 (2.13)	3 (2.8)	0 (0)	0.049 *
Middle	8 (5.67)	5 (4.67)	3 (8.82)	
Low middle	78 (55.32)	54 (50.47)	24 (70.59)	
Low	52 (36.88)	45 (42.06)	7 (20.59)	
Access to health (*n* (%))				
Patient of the social security services	55 (37.67)	43 (38.39)	12 (35.29)	0.532
Patient of Ministry of Health services	63 (43.15)	47 (41.96)	16 (47.06)	
Armed Forces/Police services	4 (2.74)	2 (1.79)	2 (5.88)	
Private health network	4 (2.74)	4 (3.57)	0 (0)	
Does not have access to any service	20 (13.7)	16 (14.29)	4 (11.76)	

* Significant differences. Source: FCM-UCE. own elaboration.

**Table 3:** Multivariable logistic regression analysis of factors associated with hearing device use.

Variable	aOR	95% CI	*p*
Microtia (familial vs. isolated)	0.42	0.13–1.35	0.14
Sex (male vs. female)	1.85	0.76–4.49	0.17
Age	0.91	0.81–1.02	0.12
SES (higher vs. lower)	2.46	0.29–20.67	0.41
Healthcare access (worse vs. better)	0.69	0.21–2.25	0.54

Note: aOR = adjusted odds ratio; CI = confidence interval. The model was adjusted for microtia type, sex, age, socioeconomic level, and healthcare access.

## Data Availability

The datasets used and/or analyzed during the current study are available from the corresponding author on reasonable request.

## Abbreviations

The following abbreviations are used in this manuscript:

BAHA	Bone-anchored hearing aid
LMICs	Low- and middle-income countries
SES	Socioeconomic status
